# Epidermal Growth Factor Mutation Analysis in Patients With Bronchogenic Adenocarcinoma: Prevalence and Clinical Profile-Outlook From a Tertiary Care Center in India

**DOI:** 10.7759/cureus.79100

**Published:** 2025-02-16

**Authors:** Guruprasad T J, K K Athish, Kavya Rani C

**Affiliations:** 1 Respiratory Medicine, Sri Devaraj Urs Medical College/R.L. Jalappa Hospital, Kolar, IND; 2 Internal Medicine, Sri Devaraj Urs Medical College/R.L. Jalappa Hospital, Kolar, IND; 3 Obstetrics and Gynecology, Sri Devaraj Urs Medical College/R.L. Jalappa Hospital, Kolar, IND

**Keywords:** bronchogenic adenocarcinoma, ecog (eastern cooperative oncology group), ecog performance status, egfr exon 19 mutation, egfr gene mutation, epidermal growth factor receptor (egfr), indian subcontinent, non-small cell lung carcinoma (nsclc), tyrosine kinase, tyrosine kinase inhibitors (tki)

## Abstract

Introduction

Epidermal growth factor receptor (EGFR) mutation analysis has become an important part of the initial workup of non-squamous non-small cell lung cancer (NS-NSCLC) patients as it is now recognized both as a prognostic and predictive marker for therapy with EGFR tyrosine kinase inhibitors (TKIs). The data on the prevalence of mutation and its clinical profile in bronchogenic adenocarcinoma are vastly available from Eastern Asian and European countries. The frequency of EGFR mutations in India however remains sparsely explored. Activating EGFR mutations in the tyrosine kinase region have been shown to underlie response to these inhibitors. However, the frequency of EGFR mutations and their clinical response in most other ethnic populations, including India, remains to be explored. In addition to providing information on the stage of the disease and the Eastern Cooperative Oncology Group (ECOG) performance scale at presentation, this is one of the rare studies from the subcontinent where EGFR mutation was performed in a single laboratory using a standardized procedure. The aims and objectives of the study are to estimate the prevalence of EGFR gene mutation in adenocarcinoma of the lung and to assess the clinical profile that correlates with EGFR gene status.

Material and methods

This single-center-based cross-sectional study was conducted at R.L. Jalappa Hospital, Kolar, India, over eight months (October 2023 to June 2024). The study included patients diagnosed with NSCLC adenocarcinoma whose participation was secured through informed consent. These tissues had been tested for EGFR mutational status. EGFR mutation analysis will be done on extracted DNA with real-time polymerase chain reaction to estimate the prevalence of EGFR mutation in adenocarcinoma of the lung. All data were entered in a Microsoft Excel sheet (Redmond, WA, USA) and statistical analysis will be performed using SPSS statistics for Windows, Version 22.0 (IBM Corp., Armonk, NY). Categorical data were represented in the form of frequencies and proportions. To check the association between qualitative data, Chi-square was applied with a level of significance defined as a p-value < 0.05. Continuous data was represented as mean and standard deviation.

Results

Of the 61 patients included in the study, the mean age of prevalence of EGFR mutation in adenocarcinoma was 58.13 years, with a prevalence rate of 31.1%. EGFR mutation was positive in 11 (42.3%) females and eight (22.8%) males. The prevalence of exon 19 deletion was the most common and was higher in females (seven (26.4%)) compared to males (eight (22.9%)). However, among those with an ECOG score of 3, one (2.3%) had EXON 18 G719S G719A G719C, and 14 (31.8%) had EXON 19 deletion. There was a significant difference (p-value<0.008) in the type of mutations concerning the ECOG performance scale.

Conclusion

The prevalence of activating EGFR mutations and their clinical correlations in our study are comparable with those previously published and Indian patients with EGFR mutations. In line with data already available from previously published studies, EGFR mutation is also a common finding in patients with lung adenocarcinoma, especially among women, and the exon 19 deletion is the most common variation. Incorporating EGFR mutation testing into early diagnostic protocols remains crucial for optimizing treatment strategies and improving patient outcomes.

## Introduction

Globally, lung cancer is the most common cause of cancer-related deaths and the most frequently diagnosed cancer. Global Cancer Observatory (GLOBOCAN) 2020 estimated that there were nearly 1.8 million lung cancer deaths (18.0%) and 2.2 million new lung cancer cases (11.4%) in 2020 [1]. As per GLOBOCAN 2022, globally the most common cancer was lung cancer with an estimated 12.4% of the total diagnosed cancer, following female breast cancers, colorectum, prostate, and stomach. Lung cancer is also the leading cause of cancer death accounting for 18.7% of the total cancer deaths. It is the most frequently diagnosed malignancy in males, lung cancer is usually associated with a positive smoking history. However, of all the lung cancers diagnosed in the world, approximately one quarter were never smokers [2], with even higher rates reported in Indian non-small cell lung cancer (NSCLC) cases (39.5%-52.7%) [3]. Adenocarcinoma, a type of NSCLC, is the most prevalent form, affecting both smokers and non-smokers [4]. This cancer often presents at an advanced stage, limiting treatment options and resulting in poor responses to standard chemotherapy and short median survival.

The epidermal growth factor receptor (EGFR) plays a role in the development of various cancers, including NSCLC [5]. EGFR, a transmembrane receptor tyrosine kinase (TK), belongs to the ErbB receptor family, which also includes HER2/neu, Her 3, and Her 4 [6]. Upon ligand binding to its extracellular domain, EGFR initiates downstream signaling pathways that promote cell growth, proliferation, and invasion [7]. Specifically, activating mutations within the EGFR TK domain predict positive responses to EGFR inhibitors, making them a valuable biomarker for patient selection in NSCLC treatment. Conversely, patients with wild-type EGFR experience less benefit from these therapies. TK domain mutations are frequently observed in lung adenocarcinoma, a characteristic targeted by TK inhibitors (TKIs) like gefitinib and erlotinib [8], which function by binding to the TK domain and disrupting cell signaling. Furthermore, the irreversible ErbB family blocker afatinib has demonstrated promising results in a phase IIb/III trial, improving progression-free survival in lung cancer patients whose disease progressed after chemotherapy and gefitinib/erlotinib treatment [9].

EGFR gene mutations in lung cancer are typically linked to adenocarcinoma histology, female gender, non-smoking status, and Asian ethnicity. These mutations occur in 10%-15% of North American and European patients, 19% of African American patients, and 20%-30% of East Asian patients, including Chinese, Korean, and Japanese individuals. However, data on EGFR mutation frequency in India are limited [10]. Exons 19-21, common mutation hotspots, are the most frequently studied, with in-frame deletions and point mutations being the most prevalent types [10]. This study aims to establish the importance of routine EGFR mutation diagnostics for NSCLC patients in India and highlight the potential of EGFR inhibitors as a first-line treatment in this population. Notably, this research is among the few from the Indian subcontinent to examine EGFR mutations using a standardized laboratory procedure, while also providing data on disease stage and the Eastern Cooperative Oncology Group (ECOG) performance status at presentation.

Objectives of the study

The objectives of this study are to estimate the prevalence of EGFR gene mutation in adenocarcinoma of the lung at a tertiary care center in India using amplification-refractory mutation system polymerase chain reaction (ARMS-PCR) analysis and to analyze the correlation between EGFR gene mutations and clinical parameters, including age, gender, smoking history, disease stage, and ECOG performance status.

## Materials and methods

This study is a single center-based cross-sectional study carried out at R.L. Jalappa Hospital, Kolar, India, for a period of eight months from October 2023 to June 2024 who were diagnosed with NSCLC-adenocarcinoma histology were included in the study, thereby eliminating the possibility of a selection bias. Possible sources of confounding that could have a bearing on the outcomes were carefully excluded during the study. Individuals with lung cancer types other than adenocarcinoma were excluded. Similarly, patients who did not consent to participate were excluded. From the included patients, genomic deoxyribonucleic acid (DNA) was extracted from the source clinical material such as tissue biopsies, formalin-fixed paraffin-embedded (FFPE) blocks, fine needle aspiration cytology (FNAC) slides, cell blocks, bronchoalveolar lavage, pleural fluid, and cell block according to standard procedures (QIAGEN DNA mini kit (ThermoFisher Scientific Inc., Waltham, MA), EGFR mutation analysis was done on extracted DNA with real-time PCR (ARMS technique). The DNA from tissue samples was isolated through a multi-step process using the QIAamp DNA Mini Kit. Initially, the tissue was lysed using Buffer ATL and Proteinase K at 56°C, which broke down cells and released DNA. Buffer AL was added, followed by incubation at 70°C, and then ethanol, which precipitated the DNA. This mixture was loaded onto a QIAamp Mini spin column. Centrifugation forced the DNA to bind to the column's silica membrane, while impurities passed through. The column was then washed with Buffer AW1 and Buffer AW2 to remove residual contaminants. High-speed centrifugation helped to eliminate any remaining Buffer AW2. Finally, the purified DNA was eluted using Buffer AE or water. This elution step involved incubating the column with the buffer and then centrifuging it to collect the DNA. Elution was repeated to increase the final DNA yield. The resulting DNA was suitable for various downstream applications. Proper reagent preparation, including adding ethanol to buffers and dissolving precipitates, was crucial. Temperature control during incubations was also essential for optimal DNA purification. Based on the previous study which showed EGFR mutation-positive in 35% of adenocarcinoma cases, with absolute precision (d) as 10% and with a 90% confidence interval (Z^2^1-α/2) the sample size was calculated using the formula, n= [Z^2^1-α/2*p*(1-p)]/d^2^, where p was expected proportion, and the minimum required sample size was 61 [11-13].

Ethical consideration

Before data collection, informed written consent was obtained from all participants. Central Ethics Committee approval (No. SDUMC/KLR/IEC/271/2023-24) was obtained before the study started at Sri Devaraj Urs Academy of Higher Education and Research, Kolar, India. The autonomy and confidentiality of the study participants were upheld throughout the research, and no participants were harmed during the study.

Statistical analysis

Data were entered into a Microsoft Excel data sheet (Redmond, WA, USA) and were analyzed using SPSS 22 version software (IBM Corp., Armonk, NY). Continuous data were expressed as mean and standard deviation. Categorical data was represented in the form of frequencies and proportions. The chi-square test was used as a test of significance for qualitative data and to test the association. Association between factors such as age, gender, smoking status, staging of lung cancer at the time of presentation, and the presence of EGFR mutation was assessed using Chi-square test and 95% confidence intervals. The P-value (probability that the result is true) of <0.05 was considered statistically significant after assuming all the rules of statistical tests.

## Results

In the study, the mean age of subjects was 58.13 ± 12.271 years. 11.5% were in the age group 20 to 40 years, 42.6% were in the age group 41 to 60 years, and 45.9% were in the age group >60 years. In the study, 42.6% were females and 57.4% were males. In the study, 82% were nonsmokers and 18% were smokers. Of the smokers, 11.5% had 10-pack years, 1.6% had 15-pack years and 4.9% had 20-pack years. On histopathological evaluation (HPE), 18% were diagnosed to have well-differentiated adenocarcinoma, 44.3% had moderately differentiated adenocarcinoma and 37.7% had poorly differentiated adenocarcinoma. 91.8% had stage IVA and 8.2% had stage IVB TNM staging (Table 1).

**Table 1:** Distribution of demographic, histopathological, and TNM staging of subjects in the study Among those with well-differentiated adenocarcinoma, 90.9% were in stage IVA and 9.1% in stage IVB. Among those with moderately differentiated adenocarcinoma, 85.2% were in stage IVA, 14.8% were in stage IVB and among those with poorly differentiated adenocarcinoma, 100% were in stage IVA. There was no significant difference in TNM staging for histopathological diagnosis. TNM: Tumor, Node, Metastasis

Characteristics	Count	%
Age		
20 to 40 years	7	11.5
41 to 60 years	26	42.6
>60 years	28	45.9
Gender		
Female	26	42.6
Male	35	57.4
Smoking		
Non-smoker	50	82.0
10-pack years	7	11.5
15-pack years	1	1.6
20-pack years	3	4.9
Histopathological diagnosis		
Well-differentiated adenocarcinoma	11	18.0
Moderately differentiated adenocarcinoma	27	44.3
Poorly differentiated adenocarcinoma	23	37.7
TNM staging at presentation		
Stage IV A	56	91.8
Stage IV B	5	8.2
Total	61	100

There was no significant difference in EXON 18 G719S G719A G719C and EXON 19 deletion mutations between males and females. There was a significant difference in EXON 21 L858R mutations between males and females (Table 2).

**Table 2:** Association of type of mutation with respect to gender Among females, 3.8% had EXON 18 G719S G719A G719C mutations, 26.9% had EXON 19 deletion, 11.5% had EXON 21 L858R mutations and none of them had EXON 20 S768I, EXON 20 insertion, EXON 20 T790M, EXON 21 L861Q mutations. Among males, 22.9% had EXON 19 deletion and none of them had EXON 18 G719S G719A G719C, EXON 20 S768I, EXON 20 insertion, EXON 20 T790M, EXON 21 L858R, and EXON 21 L861Q mutations. Among females, 3.8% had EXON 18 G719S G719A G719C mutations, 26.9% had EXON 19 deletion, 11.5% had EXON 21 L858R mutations and among males, 22.9% had EXON 19 deletion. There was no significant difference in EXON 18 G719S G719A G719C and EXON 19 deletion mutations between males and females. There was significant difference in EXON 21 L858R mutations between males and females. *P<0.05, statistically significant at 95% confidence interval, Chi-square test was used to assess the association

Mutations		Gender						P-value*
		Female		Male		Total		
		Count	%	Count	%	Count	%	
EXON 18 G719S G719A G719C	Detected	1	3.8	0	0.0	1	1.6	0.242
	Not detected	25	96.2	35	100.0	60	98.4	
EXON 19 deletion	Detected	7	26.9	8	22.9	15	24.6	0.715
	Not detected	19	73.1	27	77.1	46	75.4	
EXON 20 S768I	Detected	0	0.0	0	0.0	0	0.0	-
	Not detected	26	100.0	35	100.0	61	100.0	
EXON 20 Insertion	Detected	0	0.0	0	0.0	0	0.0	-
	Not detected	26	100.0	35	100.0	61	100.0	
EXON 20 T790M	Detected	0	0.0	0	0.0	0	0.0	-
	Not detected	26	100.0	35	100.0	61	100.0	
EXON 21 L858R	Detected	3	11.5	0	0.0	3	4.9	0.039
	Not detected	23	88.5	35	100.0	58	95.1	
EXON 21 L861Q	Detected	0	0.0	0	0.0	0	0.0	-
	Not detected	26	100.0	35	100.0	61	100.0	

In the study, there was no significant association between mutations and age distribution (Table 3).

**Table 3:** Association between various mutations and age In the study, among those in the age group 20 to 40 years, 42.9% had EXON 19 deletion, among those in the age group 41 to 60 years 15.4% had EXON 19 deletion and among those in the age group more than 60 years, 3.6% had EXON 18 G719S G719A G719C mutation, 28.6% had EXON 19 deletion and 10.7% had EXON 21 L858R mutations. *P<0.05, statistically significant at 95% confidence interval, Chi-square test was used to assess the association

Age (years)	20-40		41- 60		>60		P-value*
Mutations	Count	%	Count	%	Count	%	
Absent	4	57.1	22	84.6	16	57.1	0.212
EXON 18 G719S G719A G719C	0	0.0	0	0.0	1	3.6	
EXON 19 deletion	3	42.9	4	15.4	8	28.6	
EXON 21 L858R	0	0.0	0	0.0	3	10.7	

**Table 4:** Association between mutations and gender *P-value less than 0.05 is considered statistically significant at 95% confidence interval, Chi-square test was used to assess the association

Mutations	Count	%		Count		%	Count	%		P-value*
Gender	Female			Male			Total			
Absent	15		57.7	27	77.1		42		68.9	0.098
EXON 18 G719S G719A G719C	1		3.8	0	0.0		1		1.6	
EXON 19 deletion	7		26.9	8	22.9		15		24.6	
EXON 21 L858R	3		11.5	0	0.0		3		4.9	

Among nonsmokers, 2% had EXON 18 G719S G719A G719C mutations, 22% had EXON 19 deletion, 6% had EXON 21 L858R and among smokers, 36.4% had EXON 19 deletion. There was no significant difference in mutations between smokers and non-smokers (Table 5). On comparison of various mutations with age, gender, and smoking status (Tables 3-5), only there was a significant difference in EXON 21 L858R mutations between males and females. There was no statistically significant association between mutation and pack years (Table 6).

**Table 5:** Association between various mutations and smoking status Among nonsmokers, 2% had EXON 18 G719S G719A G719C mutations, 22% had EXON 19 deletion and 6% had EXON 21 L858R and among smokers, 36.4% had EXON 19 deletion. There was no significant difference in mutations between smokers and non-smokers. *P-value less than 0.05 is considered statistically significant at 95% confidence interval, Chi-square test was used to assess the association

Mutations	Count	%		Count		%	Count	%		P-value*
Smoking	Non-smokers			Smokers			Total			
Absent	35		70.0	7	63.6		42		68.9	0.639
EXON 18 G719S G719A G719C	1		2.0	0	0.0		1		1.6	
EXON 19 deletion	11		22.0	4	36.4		15		24.6	
EXON 21 L858R	3		6.0	0	0.0		3		4.9	

**Table 6:** Association between mutation and pack years Among those with 10-pack years, one subject had EXON 19 deletion, among those with 20-pack years, three subjects had EXON 19 deletion, and among those with 15-pack years none of them had mutations.

Mutations	Smoking								P-value
	Non-smoker		10-pack years		15-pack years		20-pack years		
	Count	%	Count	%	Count	%	Count	%	
Absent	35	70.0	6	85.7	1	100.0	0	0.0	0.275
EXON 18 G719S G719A G719C	1	2.0	0	0.0	0	0.0	0	0.0	
EXON 19 deletion	11	22.0	1	14.3	0	0.0	3	100.0	
EXON 21 L858R	3	6.0	0	0.0	0	0.0	0	0.0	

In this study, of those with ECOG score 2, 5.9% had EXON 19 deletion, 17.6% had EXON 21 L858R mutation and among those with ECOG score 3, 2.3% had EXON 18 G719S G719A G719C, 31.8% had EXON 19 deletion. There was a significant difference in the type of mutations with respect to the ECOG performance scale (Table 7).

**Table 7:** Association between mutation and ECOG performance score ECOG: Eastern Cooperative Oncology Group performance scale *P<0.05 is considered statistically significant at 95% confidence interval

Mutations	Performance scale (ECOG)				P-value*
	2		3		
	Count	%	Count	%	
Absent	13	76.5	29	65.9	0.008
EXON 18 G719S G719A G719C	0	0.0	1	2.3	
EXON 19 deletion	1	5.9	14	31.8	
EXON 21 L858R	3	17.6	0	0.0	

Among those with well-differentiated adenocarcinoma, 9.1% had EXON 19 deletion and EXON 21 L858R, respectively, among those with moderately differentiated adenocarcinoma, 3.7% had EXON 18 G719S G719A G719C, 33.3% had EXON 19 deletion and 7.4% had EXON 21 L858R respectively. Among those with poorly differentiated adenocarcinoma, 21.7% had EXON 19 deletion. There was no significant difference in mutations with respect to histopathological diagnosis (Table 8).

**Table 8:** Association between mutation and histopathological diagnosis *P<0.05 considered statistically significant at 95% confidence interval

Mutations	Histopathological diagnosis						P-value*
	Well-differentiated		Moderately differentiated		Poorly differentiated		
	Count	%	Count	%	Count	%	
Absent	9	81.8	15	55.6	18	78.3	0.383
EXON 18 G719S G719A G719C	0	0.0	1	3.7	0	0.0	
EXON 19 deletion	1	9.1	9	33.3	5	21.7	
EXON 21 L858R	1	9.1	2	7.4	0	0.0	

Among those with stage IVA, 1.8% had EXON 18 G719S G719A G719C, 25% had EXON 19 deletion and 5.4% had EXON 21 L858R mutations and among those in stage IV B, 20% had EXON 19 deletion mutations. There was no significant difference in TNM staging and type of mutations (Table 9).

**Table 9:** Association of mutation and TNM staging *P<0.05 is considered statistically significant at 95% confidence interval TNM: Tumor, Node, Metastasis

Mutations	TNM staging at presentation				P-value*
	Stage IV A		Stage IV B		
	Count	%	Count	%	
Absent	38	67.9	4	80.0	0.918
EXON 18 G719S G719A G719C	1	1.8	0	0.0	
EXON 19 deletion	14	25.0	1	20.0	
EXON 21 L858R	3	5.4	0	0.0	

## Discussion

The identification of EGFR mutations in NSCLC and the subsequent remarkable responses to TKIs represent a significant advancement in NSCLC patient care over the past decade. Numerous prospective studies have clearly shown that activating mutations within the EGFR TK domain, particularly in-frame deletions in exon 19 and the L858R missense mutation in exon 21, are strong predictors of both treatment response and survival benefit with EGFR TKIs [14,15]. Consequently, EGFR mutation status assessment has become a standard component of initial NSCLC management algorithms in many centers globally, including those in India. However, it is important to note that EGFR mutation prevalence in NSCLC varies considerably across different ethnic groups. In our study, we report a prevalence rate of 31.1% EGFR mutation in adenocarcinoma. Similarly, in a study conducted by Kota et al. [14] the prevalence of EGFR mutations was 30.6%. Zhang et al. [16] in a meta-analysis and review estimated the overall pooled prevalence for EGFR mutations as 32.3%. Chatterjee et al. [17] revealed the incidence of EGFR-positive mutation in non-small-cell lung cancer with adenocarcinoma histology as 33%. In our study, EGFR mutation was positive in 42.2% of females, and 22.9% in males. Similarly, a study from the subcontinent conducted by Kota et al. [14] reported a significantly higher rate in females (44%) as compared to men. Zhang et al. [16] reported a pooled prevalence of EGFR mutation with a higher prevalence in females (43.7%).

In our study, the prevalence of exon 19 deletion was the most common and was higher in females (26.4%) when compared to males (22.9%). Our results were similar, exon 19 deletion was common in females. But, according to Tanaka et al. [18] deletions in exon 19 were more frequently associated with the male gender while exon 21 deletions were more common among females which was exactly opposite to our results. Hence, at this moment we cannot draw a conclusion regarding which mutation is common among the genders. There may be differences between populations, as our study results were similar to those of Kota et al. [14], also a sub-continental population, and different from Tanaka et al., whose results were obtained in a Japanese population. In this study, the distribution of age did not have any significant effect on the EGFR mutation prevalence, as well as there was no difference in the type of mutation among the age groups.

The prevalence of EGFR mutation among nonsmokers is much higher as reported in the literature [19] but it was not found to be significant in our study. The probable reason for this discordance might be the fact that very few of our patient population were smokers (11/61, i.e., 19.1%). The majority of mutations were found in EXON 19 both in smokers and nonsmokers in our population. Similar results were found in a literature conducted by Kosaka et al. where exon 19 mutations were predominant among nonsmokers [20]. Also, there was no significant difference in mutation rate between heavier and lighter smokers, again reflecting perhaps the small numbers of smokers in our population. The study conducted by Shi et al. [19] had higher EGFR mutation frequency among never-smokers (60.7%) and was highest among 0-10 pack-years of smoking history (57.9%). The prevalence of different mutations among the ECOG performance scale was also done. It was found that the rate of mutation in EXON 19 was more common when the ECOG score was 3 or more whereas EXON 21 mutation was seen more when the ECOG was 2. Though no literature is available regarding these findings, it may be more useful if further study is conducted with a larger sample size.

Regarding the differentiation of adenocarcinoma, it is reported that EGFR mutations were more commonly seen in well to moderately differentiated adenocarcinoma than in poorly differentiated adenocarcinoma [21], but we did not find any significant difference in mutations with respect to differentiation of adenocarcinoma on histopathological diagnosis.

Some of the studies report that the prevalence of EGFR mutation is more common in advanced stages of adenocarcinoma particularly true in cases of extrathoracic metastasis [19], while few report that EGFR mutation occurs during the early stage of pathogenesis of adenocarcinoma hence presence of EGFR mutation does not suggest advanced diseases, which was the finding in our study also, where there was no significant association between TNM stage and mutation rate and type. Also, we cannot draw any conclusion from our sample because most of our patients were in stage 4 at the time of presentation.

Data suggest that EGFR mutation analysis should be incorporated into the early management algorithm of NS-NSCLC patients [22]. Though there was no clinical significance in the rate of mutation among nonsmokers in our study, most studies report that the prevalence is much higher among nonsmokers. Hence, it is worth screening adenocarcinoma for EGFR mutations as it has significant implications for the management of the disease [23].

The limitation of the study was, firstly considering the cross-sectional study model, the duration of the study was short. Secondly, the sample size was smaller; hence results might not have achieved statistical significance. Thirdly, most of the patients were in stage 4, hence, we could not conclude different stages and their association with EGFR mutation. Lastly, no association was studied on the radiological profile and type of metastases in these subjects. More prospective studies with larger sample sizes are required from the Indian subcontinent to understand the association of EGFR mutation concerning clinical profiles like performance status, metastasis, and response to therapy. The strength of the study is, that this is one of the few studies from the subcontinent where EGFR mutation was done in a single laboratory with a standardized procedure, which also gives the data about the stage of the disease and performance scale at presentation.

## Conclusions

In line with the data already available from previously published studies, EGFR mutation is a common finding in patients with lung adenocarcinoma, especially among women, and the exon 19 deletion is the most common variation. Testing of EGFR mutation should be incorporated into the early diagnostic algorithm of NSCLC patients for optimizing treatment strategies and improving patient outcomes, particularly with the advent of EGFR TKIs. While our study highlights a prevalence of EGFR mutations in adenocarcinoma, variations in mutation rates based on gender, smoking status, and clinical factors suggest a need for further research, particularly with larger sample sizes.
